# Allergen Immunotherapy: A Centenary Celebration

**DOI:** 10.1097/WOX.0b013e3182218920

**Published:** 2011-06-15

**Authors:** Stephen R Durham, Harold Nelson

**Affiliations:** 1 Royal Brompton National Heart & Lung Institute, London; 2 National Jewish Health, Denver, CO

## 

**Figure 1:**
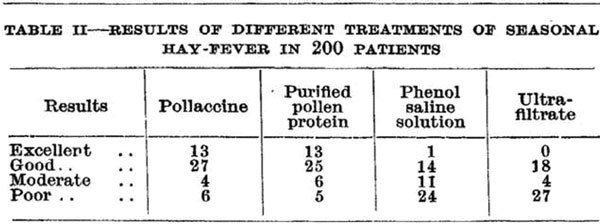
**Table II shows the results of the first immunotherapy controlled trial, as published in: Frankland AW, Augustin R**. Prophylaxis of summer hay-fever and asthma: a controlled trial comparing crude grass-pollen extracts with the isolated main protein component, *The Lancet*, May 24, 1954.

**Figure 2:**
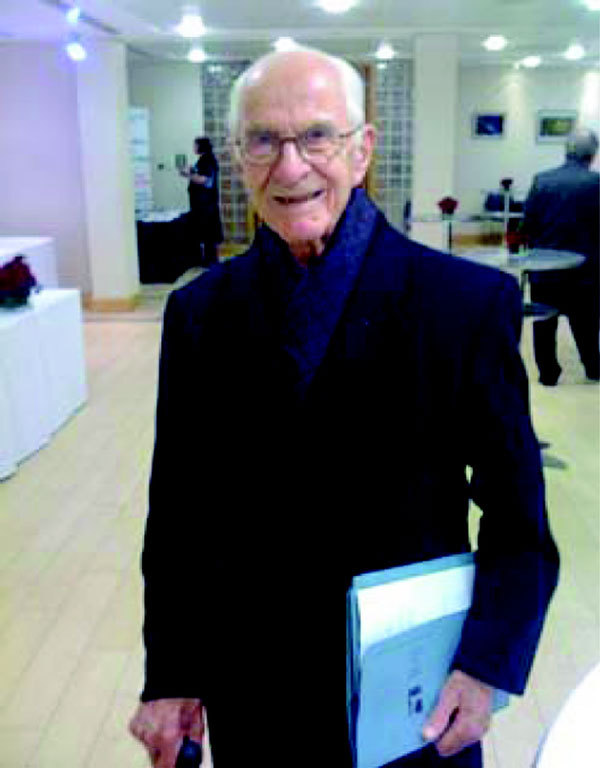
**Dr. A. William Frankland in his 99th year, our centenary link with the origins of immunotherapy**.

In 1911 and long before the availability of antiallergic drugs, Leonard Noon demonstrated that prophylactic subcutaneous inoculation with a grass pollen extract was effective in suppressing immediate conjunctival sensitivity to grass pollen [1]. Noon's coworker, John Freeman, continued to practice immunotherapy and in 1930 published the first rush immunotherapy protocol [2]. William Frankland, a colleague of Freeman, performed the first controlled clinical trial of grass pollen immunotherapy in 1954 [3]. He used a whole grass pollen extract that was compared with its partially purified proteins, the corresponding ultrafiltrate that contained no protein and a phenol-containing diluent. Both the whole extract and the purified grass pollen proteins were effective compared with the ultrafiltrate and the diluent control alone (Figure 1). Frankland's study established a firm scientific foundation for the practice of allergen immunotherapy. Noon, Freeman, and Frankland were all physicians at St. Mary's Hospital, Paddington, now affiliated to Imperial College London, United Kingdom. Frankland in his 99th year continues to practice and play a major role in the allergy community; much admired and respected by his colleagues, he represents our centenary link with the origins of the practice of immunotherapy (Figure 2).

Major advances in allergen immunotherapy have resulted from parallel studies performed in the United States. Lowell and Franklin in 1964 were the first to clearly demonstrate that a single allergen (ragweed) in a multiallergen mixture was effective in reducing seasonal allergic symptoms [4]. Philip Norman and Larry Lichtenstein in 1978 convincingly demonstrated the allergen-specificity of ragweed immunotherapy in patients with dual sensitivity to ragweed and grass pollen [5]. Johnstone first highlighted the possibility that immunotherapy in children might confer protection against development of asthma, [6] a concept supported by the more recent Preventive Allergy Treatment (PAT) study that identified a 2-3 fold risk reduction for developing asthma after 3 years treatment in children with seasonal pollinosis, protection that persisted for a further 7 years after discontinuation of immunotherapy [7]. Hunt and colleagues[8] demonstrated the efficacy of purified venom over whole insect body extract and placebo in patients with anaphylaxis to the stings of hymenoptera. Studies[9] have confirmed the dose-dependency of allergen immunotherapy whereas the long-term benefits of allergen immunotherapy, with persistence of efficacy for several years after discontinuation[10] have been illustrated for both venom[11] and grass pollen immunotherapy, the latter both for subcutaneous and sublingual routes of therapy [12].

Our increasing knowledge of the mechanisms of immunotherapy has informed both novel approaches and the development of putative biomarkers that might predict the clinical response to immunotherapy. Prausnitz and Kustner[13] published in 1921 that a serum factor ('reagin') could transfer immediate allergen sensitivity as shown by skin testing was followed Robert Cooke's observation in 1935 that serum obtained after pollen immunotherapy could confer 'immunity and hypersensitivity.'[14] These seminal observations long preceded the discovery of IgE antibody as reagin by the Ishizakas, [15] Johansson and Bennich, [16] and the concept of IgG 'blocking antibodies.'[17] The suppressive effect of ragweed immunotherapy on nasal eosinophils as a local marker of allergic inflammation was shown by Creticos in 1984, [18] whereas Passalacqua and Canonica similarly demonstrated decreased local eosinophilia and adhesion molecule expression during mite sublingual immunotherapy [19]. Warner in 1978[20] and Rak[21] in 1991 observed decreases in allergen-induced late asthmatic responses and associated bronchial inflammation, respectively, in children and adults. A link between altered T-cell responses and immunotherapy was first shown by Rocklin[22] whereas the critical role of regulatory T cells and IL-10 was highlighted by Akdis and colleagues [23]. The concept of immune deviation of allergen-specific T_H_2 responses in favor of T_H_1 responses in both the periphery and in target organs has developed in parallel [24-26].

It is paradoxical that 100 years on we continue to use conventional high-dose subcutaneous injection immunotherapy with allergen extracts as gold standard therapy. A key question remains whether either B cell and/or T cell epitopes expressed by allergens are necessary singly or together for successful immunotherapy. Whether T-cell focused therapies alone are sufficient is currently being tested in the context of T-cell peptide immunotherapy [27,28]. Other novel approaches such as the focus on adjuvants in combination with allergens, [29,30] alternative routes of administration (sublingual, [31] transdermal, [32] intranodal[33]) and the use of recombinant allergens and their hypo-allergenic variants[34] are all currently being tested in phase II-III trials.

These novel strategies will hopefully augment efficacy while improving the safety of immunotherapy, thereby making immunotherapy more broadly available to allergy sufferers, including patients with more severe allergic asthma. Further confirmation that immunotherapy has potential to induce remission and prevent progression of allergic disease should attract earlier interventions in children and young adults who are the group who may potentially benefit most from this disease-modifying treatment.
